# USP1-dependent nucleolytic expansion of PRIMPOL-generated nascent DNA strand discontinuities during replication stress

**DOI:** 10.1093/nar/gkad1237

**Published:** 2024-01-05

**Authors:** Alexandra Nusawardhana, Lindsey M Pale, Claudia M Nicolae, George-Lucian Moldovan

**Affiliations:** Department of Biochemistry and Molecular Biology, The Pennsylvania State University College of Medicine, Hershey, PA 17033, USA; Department of Biochemistry and Molecular Biology, The Pennsylvania State University College of Medicine, Hershey, PA 17033, USA; Department of Biochemistry and Molecular Biology, The Pennsylvania State University College of Medicine, Hershey, PA 17033, USA; Department of Biochemistry and Molecular Biology, The Pennsylvania State University College of Medicine, Hershey, PA 17033, USA

## Abstract

DNA replication stress-induced fork arrest represents a significant threat to genomic integrity. One major mechanism of replication restart involves repriming downstream of the arrested fork by PRIMPOL, leaving behind a single-stranded DNA (ssDNA) gap. Accumulation of nascent strand ssDNA gaps has emerged as a possible determinant of the cellular hypersensitivity to genotoxic agents in certain genetic backgrounds such as BRCA deficiency, but how gaps are converted into cytotoxic structures is still unclear. Here, we investigate the processing of PRIMPOL-dependent ssDNA gaps upon replication stress induced by hydroxyurea and cisplatin. We show that gaps generated in PRIMPOL-overexpressing cells are expanded in the 3′-5′ direction by the MRE11 exonuclease, and in the 5′-3′ direction by the EXO1 exonuclease. This bidirectional exonucleolytic gap expansion ultimately promotes their conversion into DSBs. We moreover identify the de-ubiquitinating enzyme USP1 as a critical regulator of PRIMPOL-generated ssDNA gaps. USP1 promotes gap accumulation during S-phase, and their expansion by the MRE11 and EXO1 nucleases. This activity of USP1 is linked to its role in de-ubiquitinating PCNA, suggesting that PCNA ubiquitination prevents gap accumulation during replication. Finally, we show that USP1 depletion suppresses DSB formation in PRIMPOL-overexpressing cells, highlighting an unexpected role for USP1 in promoting genomic instability under these conditions.

## Graphical Abstract

**Graphical Abstract:**
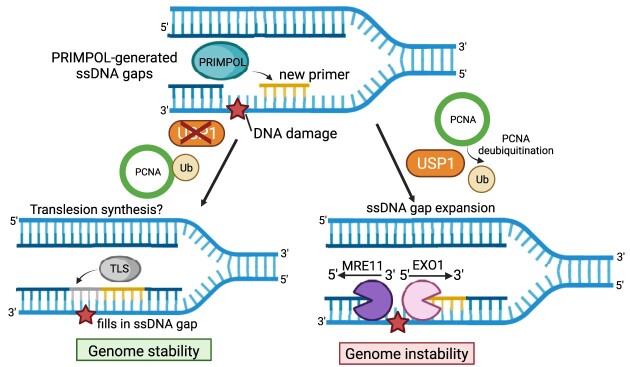

## Introduction

Exposure to genotoxic agents, including several agents used in chemotherapy such as cisplatin, creates DNA structures that can arrest the progression of the replication machinery (1–4). Replication fork progression defects can also arise from inhibition of DNA damage response and repair factors (5–9). Unless efficiently restarted, stalled replication forks can result in fork collapse and generation of double stranded DNA breaks (DSBs) (1,2). To avoid this, cells developed mechanisms to stabilize and restart the fork, thus promoting genomic stability, and, from a chemotherapy perspective, causing chemoresistance since they help the cell tolerate chemotherapy-induced DNA lesions. These mechanisms harness activities of separate DNA repair processes and coordinate them in a highly-controlled manner. In general, two main mechanisms are the most significant participants to fork protection, known as fork reversal and fork restart.

Fork reversal, a process in which the two nascent strands anneal to each other (10), allows time for DNA repair mechanisms to fix the DNA lesion, and may also eventually enable replication restart using the nascent strand of the sister chromatid as template. In this case, once DNA synthesis proceeds past the lesion, the fork can be reversed back to the original templates to resume normal replication, leaving behind the lesion to be fixed at a later time (11–13). However, fork reversal leaves nascent DNA vulnerable to nucleolytic degradation, since it exposes a double strand break (DSB) end. Reversed forks are protected by the activity of the BRCA tumor suppression pathway, and are subjected to nucleolytic cleavage by MRE11, EXO1 and other nucleases in BRCA-deficient cells (14–22).

Alternatively, stalled forks can be immediately restarted, without being subjected to reversal. Translesion synthesis (TLS) DNA polymerases are able to bypass some DNA lesions, albeit in a potentially mutagenic manner, thereby allowing replication to continue without interruption across and beyond the lesion (23,24). A separate mechanism of replication restart involves the rapid initiation of DNA synthesis downstream of the lesion to restore timely DNA replication, leaving behind a single stranded DNA (ssDNA) gap to be filled at a later stage (25–34). This is catalyzed by the PRIMPOL enzyme, which contains both primase and DNA polymerase activities within the same polypeptide (35,36). Once PRIMPOL initiates replication, it is presumably exchanged with the replicative polymerase for long-range processive DNA synthesis. Subsequently, the ssDNA gap left behind needs to be filled to restore normal DNA structure. Indeed, accumulation of nascent strand ssDNA gaps was proposed as a major determinant of the cellular hypersensitivity to genotoxic agents (including chemotherapies such as cisplatin and PARP1 inhibitors) (4,8,26,29,37–47).

Recent studies have mapped two main mechanisms of ssDNA gap repair (4,8,26,29,37–51): One involves homology-dependent gap filling, using the nascent strand of the sister chromatid, through either template switching or BRCA-mediated homologous recombination repair. The other one entails gap filling by TLS polymerases, such as the REV1- Polς complex and the Polθ polymerase-helicase. In BRCA-deficient cells, TLS becomes critical for gap filling.

How ssDNA gaps promote cellular toxicity is unclear. In BRCA-deficient cells, ssDNA gaps were shown to be expanded by the MRE11 nuclease (29,45). MRE11 possesses both endonuclease and 3′-5′ exonuclease activities. If ssDNA gaps are also extended in the 5′-3′ direction is unclear. Importantly, ssDNA accumulation has been associated with formation of cytotoxic DSBs, although it is a matter of debate if these DSBs are formed directly from the ssDNA gaps or arise subsequently during apoptosis (8,37,39,43). Moreover, the relationship between gap expansion by exonucleases and DSB formation is also unclear.

TLS is regulated through the ubiquitination of the homotrimeric ring-shaped protein Proliferating Cell Nuclear Antigen (PCNA), an essential component of the replication machinery which encircles and slides along the DNA during DNA synthesis (52–54). PCNA interacts with the replicative polymerases on each strand and enhances their processivities. At stalled replication forks, PCNA mono-ubiquitination promotes the switch from the replicative polymerase to TLS polymerases. RAD18 is the major E3 ubiquitin ligase for PCNA ubiquitination, while USP1 was shown to promote its de-ubiquitination (55,56). In respect to ssDNA gap repair, RAD18-mediated PCNA ubiquitination was shown to promote gap filling in G2 (29). While USP1 inhibition was found to be toxic in BRCA-deficient cells due to increased replication stress (57,58), whether PCNA de-ubiquitination plays a role in gap accumulation remains unclear.

Here, we investigate the processing of PRIMPOL-generated ssDNA gaps upon replication stress induced by hydroxyurea (HU) and cisplatin. By using PRIMPOL-overexpressing HeLa and U2OS cells, we show that ssDNA gaps generated under these conditions are expanded in the 3′-5′ direction by the MRE11 exonuclease, and in the 5′-3′ direction by the EXO1 exonuclease. We moreover show that this bidirectional exonucleolytic processing of ssDNA gaps ultimately promotes their conversion into DSBs. In addition, we identify USP1 as a critical regulator of the metabolism of PRIMPOL-generated ssDNA gaps. We show that USP1 promotes the accumulation of ssDNA gaps during S-phase, and their expansion by the MRE11 and EXO1 nucleases. Importantly, this activity of USP1 is linked to its role in de-ubiquitinating PCNA, suggesting that PCNA ubiquitination participates in suppressing gap accumulation during replication. Finally, we show that USP1 depletion prevents DSB formation in PRIMPOL-overexpressing cells, highlighting an unexpected role for USP1 in promoting genomic instability under these conditions.

## Materials and methods

### Cell culture and protein techniques

HeLa and U2OS cells, obtained from ATCC, were grown in Dulbecco's modified Eagle's media (DMEM) supplemented with 15% FBS and penicillin/streptomycin. For PRIMPOL overexpression, the pLV[Exp]-Hygro-CMV > hPRIMPOL lentiviral construct (Cyagen) was used. Infected cells were selected by hygromycin.

Gene knockdown was performed using Lipofectamine RNAiMAX. AllStars Negative Control siRNA (Qiagen 1027281) was used as control. The following oligonucleotide sequences (Stealth or SilencerSelect siRNA, ThermoFisher) were used:

MRE11: AAUAACUCGAGGCAGGUAUGUAAUG;

EXO1: CCUGUUGAGUCAGUAUUCUCUUUCA;

RAD18: Assay ID s32295;

USP1#1: Assay ID s14724 (used unless otherwise specified);

USP1#2: Assay ID s14725;

MRE11#2: AGAAACAUGUUGGUUUGCUGCGUAU;

EXO1#2: Assay ID s17503;

REV1: GAAAUCCUUGCAGAGACCAAACUUA.

Denatured whole cell extracts were prepared by boiling cells in 100mM Tris, 4% SDS, 0.5M β-mercaptoethanol. Antibodies used for Western blot, at 1:500 dilution, were:

MRE11 (GeneTex GTX70212);

EXO1 (Novus NBP2-16391);

PRIMPOL (Proteintech 29824–1-AP);

RAD18 (Cell Signaling Technology 9040);

USP1 (Abcam ab264221);

Ubiquityl-PCNA Lys164 (Cell Signaling Technology 13439);

REV1 (Santa Cruz Biotechnology sc-393022);

GAPDH (Santa Cruz Biotechnology sc-47724);

Vinculin (Santa Cruz Biotechnology sc-73614).

USP1 inhibitors used were ML323 (MedChem Express, HY-17543) and KSQ-4279 (MedChem Express, HY-145471). MRE11 inhibitors used were the exonuclease inhibitor mirin (Selleck Chemicals S8096) and the endonuclease inhibitor PFM01 (Tocris 6222).

### Functional assays

Neutral and BrdU alkaline comet assays were performed as previously described (47) using the Comet Assay Kit (Trevigen, 4250-050). For the BrdU alkaline comet assay, cells were incubated with 100μM BrdU as indicated. Chemical compounds (HU, cisplatin, ML323) were added according to the labeling schemes presented. Slides were stained with anti-BrdU (BD 347580) antibodies and secondary AF568-conjugated antibodies (Invitrogen A-11031), and imaged on a Nikon microscope operating the NIS Elements V1.10.00 software. Olive tail moment was analyzed using CometScore 2.0. Immunofluorescence was performed as previously described (59) using a γH2AX antibody (MilliporeSigma JBW301). Slides were imaged on a confocal microscope (Leica SP5) and analyzed using ImageJ 1.53a software.

### S1 nuclease DNA fiber combing assays

Cells were incubated with 100 μM IdU and 100μM CldU as indicated. Chemical compounds (HU, cisplatin, ML323) were added according to the labeling schemes presented. Next, cells were collected and processed using the FiberPrep kit (Genomic Vision EXT-001) according to the manufacturer's instructions. Samples were added to combing reservoirs containing MES solution (2-(*N*-morpholino) ethanesulfonic acid) supplemented with 1 mM zinc acetate and either 40 U/ml S1 nuclease (ThermoFisher 18001016) or S1 nuclease dilution buffer as control, and incubated for 30 min at room temperature. Next, DNA molecules were stretched onto coverslips (Genomic Vision COV-002-RUO) using the FiberComb Molecular Combing instrument (Genomic Vision MCS-001). Slides were then stained with antibodies detecting CldU (Abcam 6236) and IdU (BD 347580), and incubated with secondary Cy3 (Abcam 6946) or Cy5 (Abcam 6565) conjugated antibodies. Finally, the cells were mounted onto coverslips and imaged using a confocal microscope (Leica SP5) and analyzed using LASX 3.5.7.23225 software.

### SIRF assays

Cells were seeded into 8-chamber slides and 24 h later they were pulse-labeled with 50 μM EdU and treated with 0.4mM HU according to the labeling schemes presented. Cells were permeabilized with 0.5% Triton for 10 min at 4°C, washed with PBS, fixed at room temperature with 4% paraformaldehyde in PBS for 10min, washed again in PBS, and then blocked in 3% BSA in PBS for 30min. Cells were then subjected to Click-iT reaction with biotin-azide using the Click-iT Cell Reaction Buffer Kit (ThermoFisher C10269) for 30 min and incubated overnight at 4°C with primary antibodies diluted in PBS with 1% BSA. The primary antibodies used were: Biotin (mouse: Jackson ImmunoResearch 200–002-211; rabbit: Bethyl Laboratories A150-109A); MRE11 (GeneTex GTX70212); EXO1 (Santa Cruz Biotechnology sc-56092). Next, samples were subjected to a proximity ligation reaction using the Duolink kit (MilliporeSigma DUO92008) according to the manufacturer's instructions. Slides were imaged using a confocal microscope (Leica SP5) and images were analyzed using ImageJ 1.53a software. To account for variation in EdU uptake between samples, for each sample, the number of protein-biotin foci were normalized to the average number of biotin–biotin foci for that respective sample.

### Statistics and reproducibility

For SIRF assays, the t-test (two-tailed, unpaired) was used. For DNA fiber assays, immunofluorescence and comet assays the Mann–Whitney statistical test (two-tailed) was performed. For DNA fiber combing, SIRF, and comet assays, results from one experiment are shown; the results were reproduced in at least one additional independent biological conceptual replicate. Statistical analyses were performed using GraphPad Prism 10. Statistical significance is indicated for each graph (ns = not significant, for *P*> 0.05; * for *P* ≤ 0.05; ** for *P* ≤ 0.01; *** for *P* ≤ 0.001, **** for *P* ≤ 0.0001).

## Results

### PRIMPOL-generated ssDNA gaps are processed by EXO1

**Figure 1.**
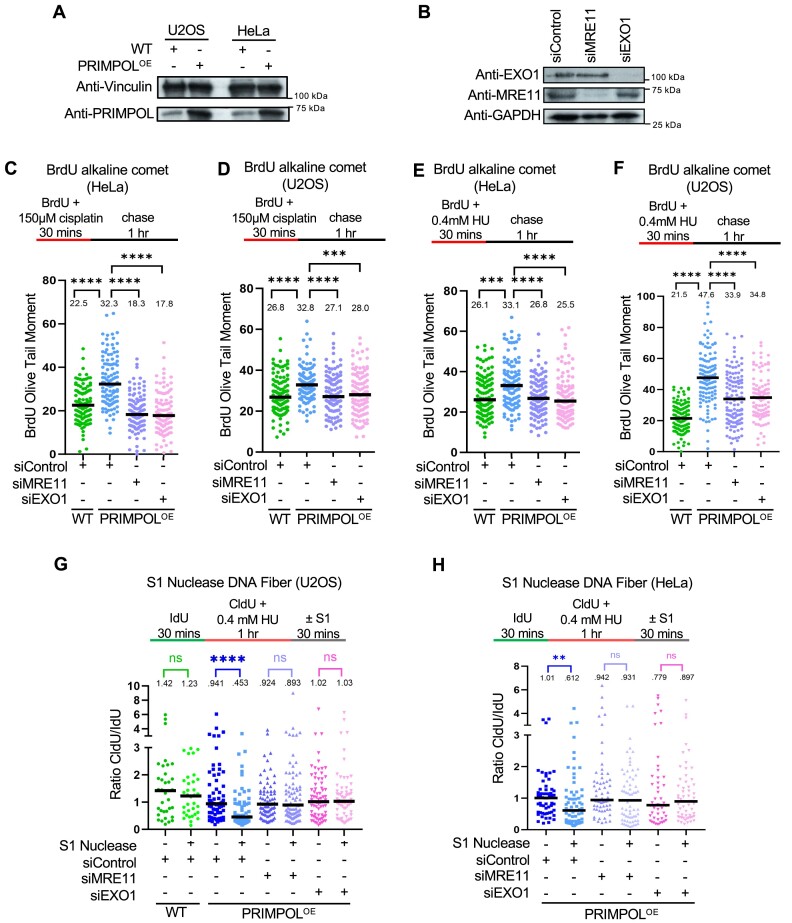
Loss of EXO1 suppresses the accumulation of nascent strand ssDNA gaps induced by HU and cisplatin in PRIMPOL-overexpressing cells. (**A**) Western blots showing the expression of PRIMPOL in control and PRIMPOL-overexpressing HeLa and U2OS cells. (**B**) Western blots showing siRNA-mediated knockdown of EXO1 and MRE11 in HeLa-PRIMPOL^OE^ cells. (C–F) BrdU alkaline comet assay showing that knockdown of EXO1 suppresses the accumulation of replication-associated ssDNA gaps induced by treatment with 150μM cisplatin (**C, D**) or 0.4mM HU (**E, F**) in HeLa (**C, E**) and U2OS (**D, F**) PRIMPOL-overexpressing cells, similar to MRE11 depletion. At least 100 nuclei were quantified for each condition. The median values are marked on the graph and listed at the top. Asterisks indicate statistical significance (Mann-Whitney, two-tailed). Schematic representations of the assay conditions are shown at the top. (G, H) S1 nuclease DNA fiber combing assays showing that knockdown of EXO1 suppresses the accumulation of nascent strand ssDNA gaps induced by treatment with 0.4mM HU in U2OS (**G**) and HeLa (**H**) PRIMPOL-overexpressing cells, similar to MRE11 depletion. The ratio of CldU to IdU tract lengths is presented, with the median values marked on the graphs and listed at the top. At least 35 tracts were quantified for each sample. Asterisks indicate statistical significance (Mann-Whitney, two-tailed). Schematic representations of the assay conditions are shown at the top.

In order to investigate the processing of nascent strand ssDNA gaps, we first sought to create a genetic system which predisposes to accumulation of ssDNA gaps in an otherwise DNA repair-proficient background. To this end, we overexpressed PRIMPOL under the control of a strong promoter, namely the CMV promoter, in both HeLa and U2OS cells (Figure 1A; Supplementary Figure S1). We next measured nascent strand ssDNA gap accumulation upon exposure to replication stress-inducing agents using the BrdU alkaline comet assay, as previously employed by us and others (44,60,61). In line with previous findings (26,29), we observed that cisplatin treatment (150 μM for 30 min) causes increased accumulation of ssDNA gaps in PRIMPOL-overexpressing (PRIMPOL^OE^) cells compared to control cells, in both HeLa and U2OS cell lines (Figure 1B–D). Recent studies found that inhibition of the MRE11 3′–5′ exonuclease activity suppresses nascent strand ssDNA gap accumulation in BRCA-deficient cells (29,45), suggesting that MRE11 exonuclease extends ssDNA gaps if they are not fixed in a timely manner. In line with this, MRE11 depletion in PRIMPOL-overexpressing cells reduced ssDNA gap accumulation in both HeLa and U2OS cells (Figure 1B–D).

In addition to MRE11, another protein with exonuclease activity involved in processing of stalled replication forks is EXO1. In contrast to MRE11, EXO1 has 5′-3′ exonuclease activity, and we recently showed that in BRCA-deficient cells, MRE11 and EXO1 cooperate upon fork reversal to extend a ssDNA nick in the nascent strand in both 5′ and 3′ directions (45). This prompted us to investigate if EXO1 is also involved in the processing of PRIMPOL-generated ssDNA gaps. Knockdown of EXO1 resulted in suppression of cisplatin-induced ssDNA gaps in both HeLa-PRIMPOL^OE^ and U2OS-PRIMPOL^OE^ cells, similar to MRE11 depletion (Figure 1B-D).

We next sought to test if this phenomenon is restricted to cisplatin-induced gaps, or also occurs in response to other types of replication stress. We previously showed that treatment of BRCA-deficient cells with a low dose of hydroxyurea (0.4 mM HU for 30mins) results in accumulation of ssDNA gaps detectable by the BrdU alkaline comet assay (44). We observed that this treatment condition also causes accumulation of gaps in BRCA-proficient, PRIMPOL-overexpressing HeLa and U2OS cells (Figure 1E, F; Supplementary Figure S2). Similar to the results obtained with cisplatin treatment described above, depletion of MRE11 or EXO1 suppressed the accumulation of ssDNA gaps induced by HU treatment in these cells (Figure 1E, F).

Finally, we also measured ssDNA gaps using an independent method to measure nascent strand ssDNA gaps, namely the S1 nuclease DNA fiber combing assay (62), to validate these findings. Treatment with 0.4 mM HU resulted in nascent strand ssDNA gaps in PRIMPOL-overexpressing U2OS and HeLa cells, as evidenced by the shortening of the CldU/IdU ratio in S1-treated samples compared to untreated samples, but not in wildtype cells (Figure 1G,H). In line with the results obtained using the BrdU alkaline comet assay, gap accumulation was suppressed by depletion of MRE11 or EXO1 using two different siRNA oligonucleotides, or inhibition of MRE11 exonuclease activity using the specific inhibitor mirin (Figure 1G, H; Supplementary Figure S3). Overall, these findings indicate that both the MRE11 3′-5′ exonuclease and the EXO1 5′-3′ exonuclease are required for ssDNA gap accumulation upon PRIMPOL overexpression.

### Loss of USP1 suppresses ssDNA gap accumulation during DNA replication

Previous studies have shown that, in BRCA-deficient cells, PRIMPOL-generated gaps can be repaired by a REV1-dependent mechanism which requires RAD18-mediated PCNA ubiquitination (28). Moreover, using an assay which measures gap filling behind the replication fork, it was shown that RAD18-mediated PCNA ubiquitination promotes the repair of cisplatin-induced ssDNA gaps generated by PRIMPOL overexpression, during the G2 phase of the cell cycle, while S-phase mediated filling of these gaps occurs through RAD51, UBC13 and REV1 but presumably independent of PCNA ubiquitination (29). On the other hand, increased PCNA ubiquitination upon inhibition of the USP1 de-ubiquitinating enzyme was found to be toxic in BRCA-deficient cells due to increased replication stress (57,58). If this reflects the role of PCNA ubiquitination in ssDNA gap metabolism is unclear.

**Figure 2.**
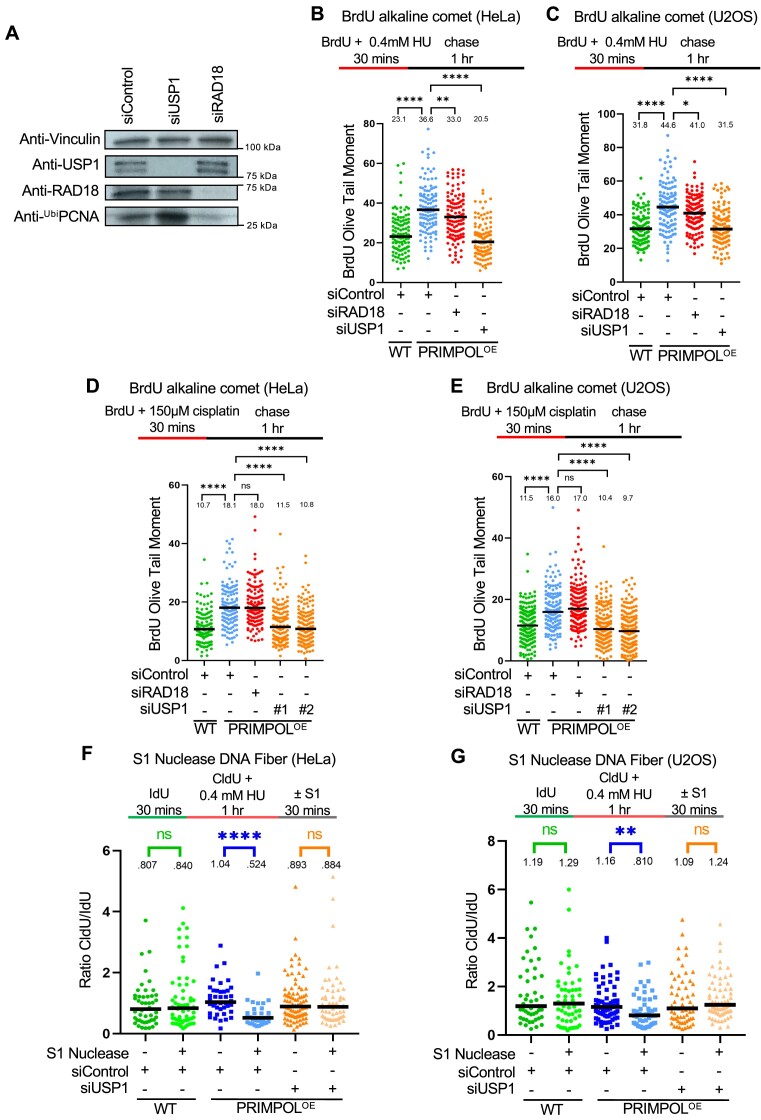
Loss of USP1 suppresses the accumulation of nascent strand ssDNA gaps induced by HU and cisplatin in PRIMPOL-overexpressing cells. (**A**) Western blots showing siRNA-mediated knockdown of RAD18 and USP1 in HeLa-PRIMPOL^OE^ cells, and the impact on PCNA ubiquitination, using an antibody that specifically recognizes mono-ubiquitinated PCNA. (B–E) BrdU alkaline comet assay showing that knockdown of USP1 suppresses the accumulation of replication-associated ssDNA gaps induced by treatment with 0.4 mM HU (**B, C**) or 150 μM cisplatin (**D, E**) in HeLa (**B, D**) and U2OS (**C, E**) PRIMPOL-overexpressing cells. At least 100 nuclei were quantified for each condition. The median values are marked on the graph and listed at the top. Asterisks indicate statistical significance (Mann–Whitney, two-tailed). Schematic representations of the assay conditions are shown at the top. (F, G) S1 nuclease DNA fiber combing assays showing that knockdown of USP1 suppresses the accumulation of nascent strand ssDNA gaps induced by treatment with 0.4 mM HU in HeLa (**F**) and U2OS (**G**) PRIMPOL-overexpressing cells. The ratio of CldU to IdU tract lengths is presented, with the median values marked on the graphs and listed at the top. At least 30 tracts were quantified for each sample. Asterisks indicate statistical significance (Mann-Whitney, two-tailed). Schematic representations of the assay conditions are shown at the top.

To address this, we depleted RAD18 or USP1 in PRIMPOL-overexpressing cells to suppress and respectively enhance PCNA ubiquitination (Figure 2A). Somewhat surprisingly, loss of RAD18 did not lead to an increase in ssDNA gaps measured by the BrdU alkaline comet assay in HeLa or U2OS PRIMPOL-overexpressing cells, upon either HU or cisplatin treatment (Figure 2B–E). This can potentially be reconciled by the previously-published observation mentioned above, that RAD18-mediated PCNA ubiquitination fills ssDNA gaps in G2 (since the experimental conditions employed in our assay are likely to mainly measure gaps in S-phase). However, loss of USP1 unexpectedly suppressed ssDNA gap accumulation in PRIMPOL-overexpressing cells under these conditions (Figure 2B-E). This was confirmed using two different siRNA oligonucleotides (Figure 2D, E). Moreover, we obtained similar results when measuring ssDNA gaps using the S1 nuclease DNA fiber combing assay, in both HeLa and U2OS cells (Figure 2F, G; Supplementary Figure S3). These findings suggest that USP1 promotes the accumulation of PRIMPOL-derived ssDNA gaps concomitant with DNA replication, potentially arguing that hyper-ubiquitination of PCNA mediates gap filling during S-phase.

### USP1 promotes the engagement of MRE11 and EXO1 nucleases for ssDNA gap expansion

**Figure 3.**
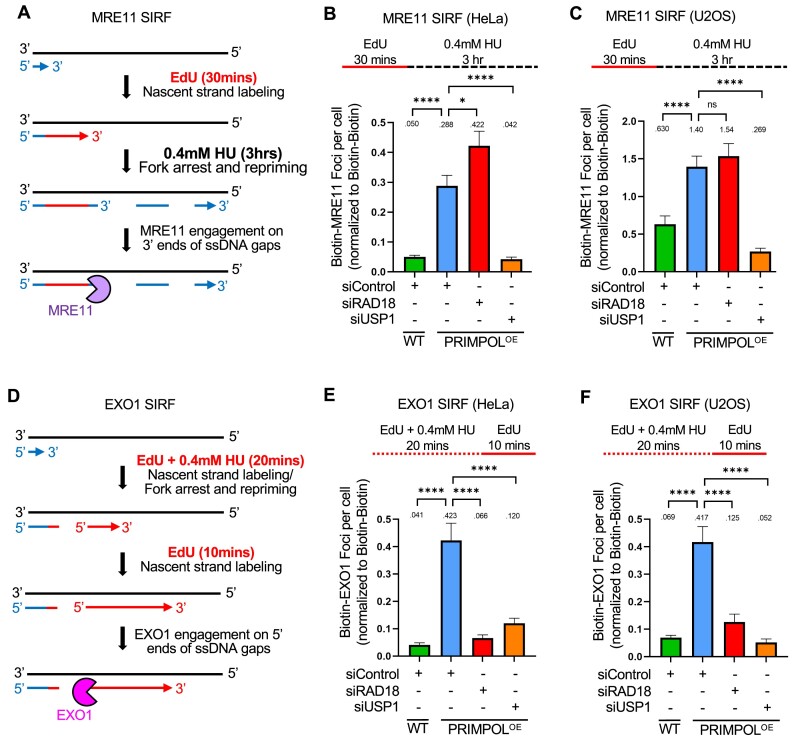
USP1 depletion suppresses the binding of MRE11 and EXO1 to replication stress-induced nascent strand ssDNA gaps in PRIMPOL-overexpressing cells. (A–C) SIRF experiments showing that treatment with 0.4mM HU induces binding of MRE11 to nascent DNA in HeLa (**B**) and U2OS (**C**) PRIMPOL-overexpressing cells, and this binding is suppressed by USP1 depletion. The labeling scheme (**A**) is designed to capture MRE11 binding to the 3′ end of the gap (for simplicity, only one strand, e.g. the leading strand, is shown in the schematic representation; EdU-labeled nascent DNA is indicated in red). At least 100 cells were quantified for each condition. Bars indicate the mean values, error bars represent standard errors of the mean, and asterisks indicate statistical significance (*t*-test, two-tailed, unpaired). Schematic representations of the assay conditions are shown at the top. (**D–F**) SIRF experiments showing that treatment with 0.4mM HU induces binding of EXO1 to nascent DNA in HeLa (E) and U2OS (F) PRIMPOL-overexpressing cells, and this binding is suppressed by USP1 depletion. The labeling scheme (D) is designed to capture EXO1 binding to the 5′ end of the gap (for simplicity, only one strand, e.g. the leading strand, is shown in the schematic representation; EdU-labeled nascent DNA is indicated in red). At least 100 cells were quantified for each condition. Bars indicate the mean values, error bars represent standard errors of the mean, and asterisks indicate statistical significance (t-test, two-tailed, unpaired). Schematic representations of the assay conditions are shown at the top.

Since the findings described above indicated that MRE11 and EXO1 expand PRIMPOL-generated ssDNA gaps, we sought to investigate if they directly engage gapped DNA under these conditions. To this end, we employed the proximity ligation (PLA)-based SIRF (*in situ* quantification of proteins interactions at DNA replication forks) assay to detect nuclease binding to EdU-labeled nascent DNA (63). Since MRE11 has 3′–5′ exonuclease activity, we first labeled cells with EdU for 30mins, then washed it away and added 0.4 mM HU for 3 h in order to measure MRE11 engagement on the 3′ end of the gap (Figure 3A). In both HeLa and U2OS cells, we observed increased binding of MRE11 to nascent DNA in PRIMPOL-overexpressing cells compared to control cells under these conditions (Figure 3B,C). Depletion of RAD18 did not affect MRE11 recruitment, but depletion of USP1 suppressed it. These findings are in line with the suppression of ssDNA gap accumulation upon USP1 depletion observed above (Figure 2).

We next investigated EXO1 recruitment to gapped DNA by SIRF. Since EXO1 exonuclease activity operates in the opposite direction than that of MRE11, we instead labeled the 5′ end of the gap by treating cells with 0.4 mM HU for 20mins, followed by EdU labeling for 10mins (Figure 3D). Using these experimental conditions, we found that EXO1 is specifically recruited to nascent DNA in PRIMPOL-overexpressing HeLa and U2OS cells (Figure 3E, F). Similar to the situation for MRE11 reported above, loss of USP1 suppressed EXO1 recruitment to nascent DNA under these conditions. Of note, unlike the case for MRE11, depletion of RAD18 surprisingly also suppressed EXO1 engagement. Overall, these findings show that, in the presence of USP1, MRE11 and EXO1 nucleases expand ssDNA gaps generated by PRIMPOL, suggesting a role for PCNA hyper-ubiquitination in restraining the activities of these nucleases in gap expansion.

### USP1 de-ubiquitination activity suppresses TLS-mediated gap filling

**Figure 4.**
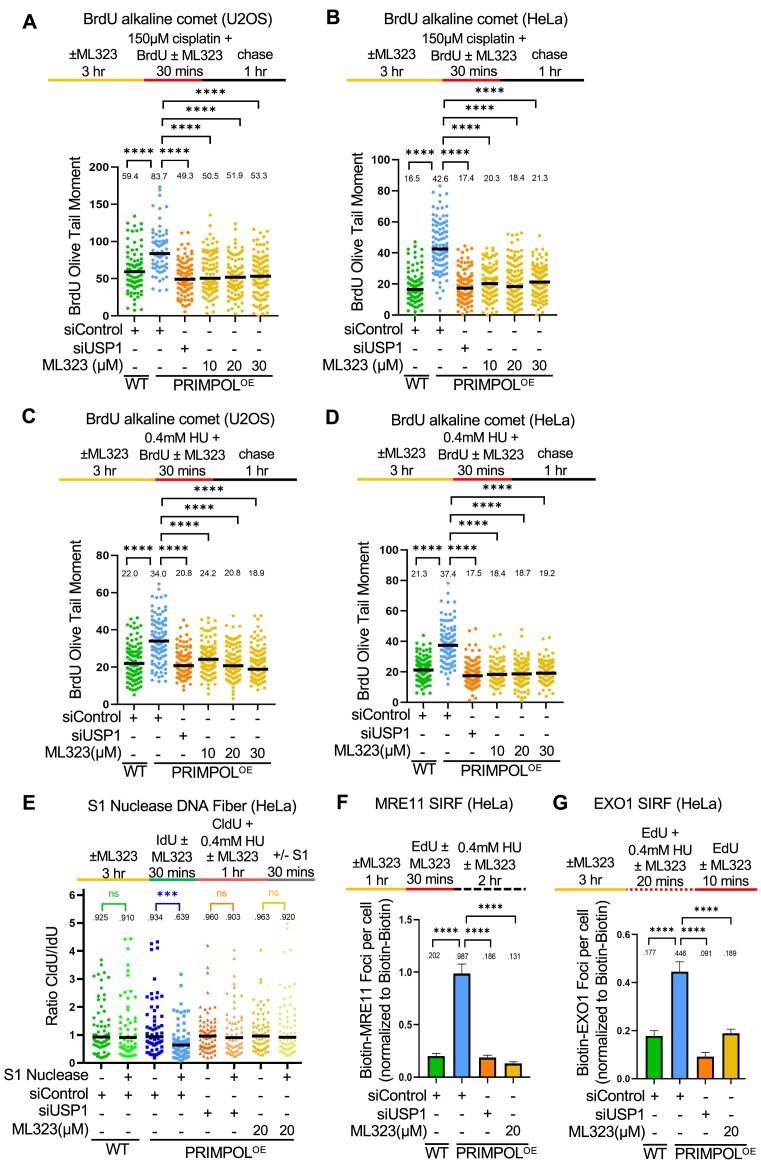
Loss of USP1 suppresses the accumulation of nascent strand ssDNA gaps induced by HU and cisplatin in PRIMPOL-overexpressing cells. (**A-D**) BrdU alkaline comet assay showing that inhibition of USP1 by treatment with ML323 as indicated, suppresses the accumulation of replication-associated ssDNA gaps induced by exposure to 150μM cisplatin (**A, B**) or 0.4mM HU (**C, D**) in U2OS (A, C) and HeLa (B, D) PRIMPOL-overexpressing cells. At least 70 nuclei were quantified for each condition. The median values are marked on the graph and listed at the top. Asterisks indicate statistical significance (Mann–Whitney, two-tailed). Schematic representations of the assay conditions are shown at the top. (**E**) S1 nuclease DNA fiber combing assay showing that inhibition of USP1 by treatment with ML323 as indicated suppresses the accumulation of replication-associated ssDNA gaps induced by exposure to 0.4 mM HU in HeLa PRIMPOL-overexpressing cells. The ratio of CldU to IdU tract lengths is presented, with the median values marked on the graphs and listed at the top. At least 50 tracts were quantified for each sample. Asterisks indicate statistical significance (Mann–Whitney, two-tailed). A schematic representation of the assay conditions is shown at the top. (F, G) SIRF experiments showing that USP1 inhibition by ML323 treatment as indicated suppresses the binding of MRE11 (**F**) and EXO1 (**G**) to nascent DNA in HeLa PRIMPOL-overexpressing cells, similar to USP1 depletion. At least 100 cells were quantified for each condition. Bars indicate the mean values, error bars represent standard errors of the mean, and asterisks indicate statistical significance (t-test, two-tailed, unpaired). Schematic representations of the assay conditions are shown at the top.

We next investigated if the role of USP1 in promoting ssDNA gap expansion by MRE11 and EXO1 described here, is dependent on its de-ubiquitination activity. To this end, we employed ML323, a selective, allosteric inhibitor of the USP1-UAF1 de-ubiquitinase complex (64). Similar to USP1 knockdown, USP1 inhibition by ML323 suppressed ssDNA gap accumulation measured by the BrdU alkaline comet assay in PRIMPOL-overexpressing HeLa and U2OS cells, upon either cisplatin (Figure 4A, B) or HU (Figure 4C, D) treatment. This was confirmed using the S1 nuclease DNA fiber combing assay in HeLa-PRIMPOL^OE^ cells (Figure 4E). Finally, we validated these results using another USP1 small molecule inhibitor, namely KSQ-4279, which selectively inhibits USP1 activity (65–67). Treatment with KSQ-4279 also suppressed ssDNA gap accumulation in PRIMPOL-overexpressing HeLa cells upon both cisplatin and HU treatment as measured by the BrdU alkaline comet assay, in a dose-dependent manner (Supplementary Figure S4A, B). This suppression was also confirmed using the S1 nuclease DNA fiber combing assay to measure HU-induced ssDNA gaps in HeLa-PRIMPOL^OE^ cells (Supplementary Figure S4C). These findings indicate that the USP1 catalytic activity promotes ssDNA gap accumulation in PRIMPOL-overexpressing cells.

We also investigated the recruitment of MRE11 and EXO1 exonucleases to nascent strand ssDNA gaps by SIRF, using the respective labeling schemes described above (Figure 3). Similar to USP1 knockdown, inhibition of USP1 by ML323 suppressed MRE11 and EXO1 engagement on PRIMPOL-generated ssDNA gaps (Figure 4F,G). These findings are in line with the observed suppression of ssDNA gap accumulation by USP1 inhibition, and indicate that the de-ubiquitination activity of USP1 promotes excessive engagement of MRE11 and EXO1 nucleases for bidirectional ssDNA gap expansion.

**Figure 5.**
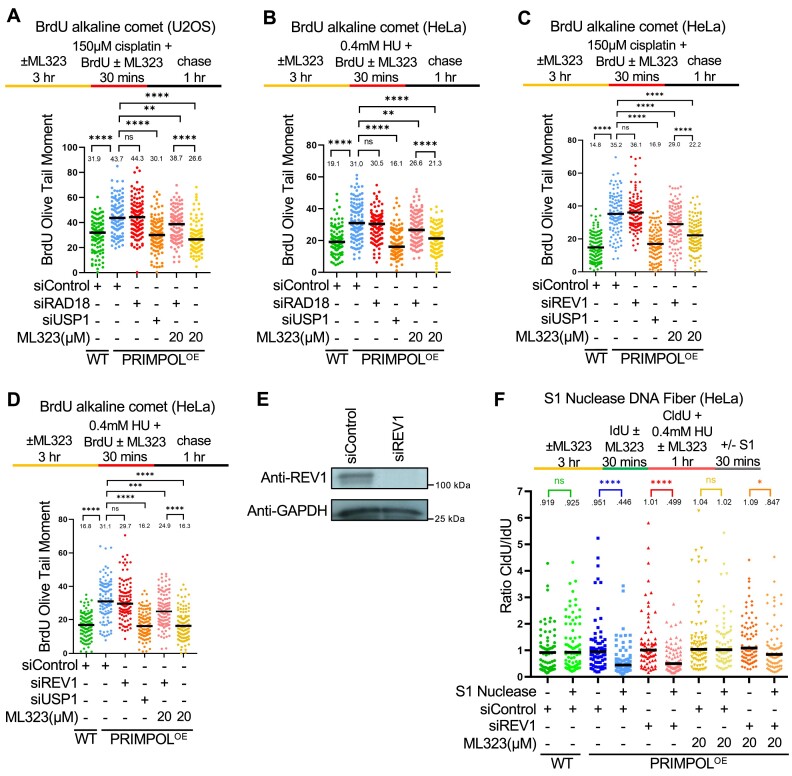
Inhibition of USP1 de-ubiquitination activity promotes TLS-mediated gap filling in PRIMPOL-overexpressing cells. (A, B) BrdU alkaline comet assays showing that RAD18 depletion reverses the suppression of ssDNA gap accumulation caused by inhibition of USP1 in U2OS (**A**) and HeLa (**B**) PRIMPOL-overexpressing cells upon treatment with cisplatin or HU as indicated. At least 100 nuclei were quantified for each condition. The median values are marked on the graph and listed at the top. Asterisks indicate statistical significance (Mann-Whitney, two-tailed). Schematic representations of the assay conditions are shown at the top. (C, D) BrdU alkaline comet assays showing that REV1 depletion reverses the suppression of ssDNA gap accumulation caused by inhibition of USP1 in HeLa PRIMPOL-overexpressing cells upon treatment with cisplatin (**C**) or HU (**D**) as indicated. At least 100 nuclei were quantified for each condition. The median values are marked on the graph and listed at the top. Asterisks indicate statistical significance (Mann-Whitney, two-tailed). Schematic representations of the assay conditions are shown at the top. (**E**) Western blots showing siRNA-mediated knockdown of REV1 in HeLa-PRIMPOL^OE^ cells. (**F**) S1 nuclease DNA fiber combing assay showing that depletion of REV1 partially restores HU-induced ssDNA gap accumulation in HeLa PRIMPOL-overexpressing cells treated with the USP1 inhibitor ML323. The ratio of CldU to IdU tract lengths is presented, with the median values marked on the graphs and listed at the top. At least 70 tracts were quantified for each sample. Asterisks indicate statistical significance (Mann-Whitney, two-tailed). A schematic representation of the assay conditions is shown at the top.

We next sought to investigate if PCNA is the relevant USP1 substrate responsible for the observed USP1-mediated gap accumulation. Depletion of RAD18 restored ssDNA gap accumulation in PRIMPOL-overexpressing cells treated with the USP1 inhibitor ML323 (Figure 5A, B), arguing that the effect of USP1 inhibition on gap suppression depends on the ability to ubiquitinate PCNA.

Since PCNA ubiquitination promotes TLS, we next investigated the possible involvement of TLS polymerases in gap suppression upon USP1 inhibition. The TLS polymerase REV1 was previously involved in gap filling (28,29). BrdU alkaline comet assays indicated that, upon either HU or cisplatin treatment, REV1 depletion was able to partially restore ssDNA gap accumulation in PRIMPOL-overexpressing HeLa cells treated with the USP1 inhibitor ML323 (Figure 5C–E). Similar results were obtained when employing the S1 nuclease DNA fiber combing assay (Figure 5F). Overall, these findings argue that USP1-mediated PCNA de-ubiquitination is responsible for promoting gap expansion during DNA replication, by suppressing the recruitment of TLS polymerases.

### Expansion of ssDNA gaps by MRE11, EXO1 and USP1 results in accumulation of double strand breaks

**Figure 6.**
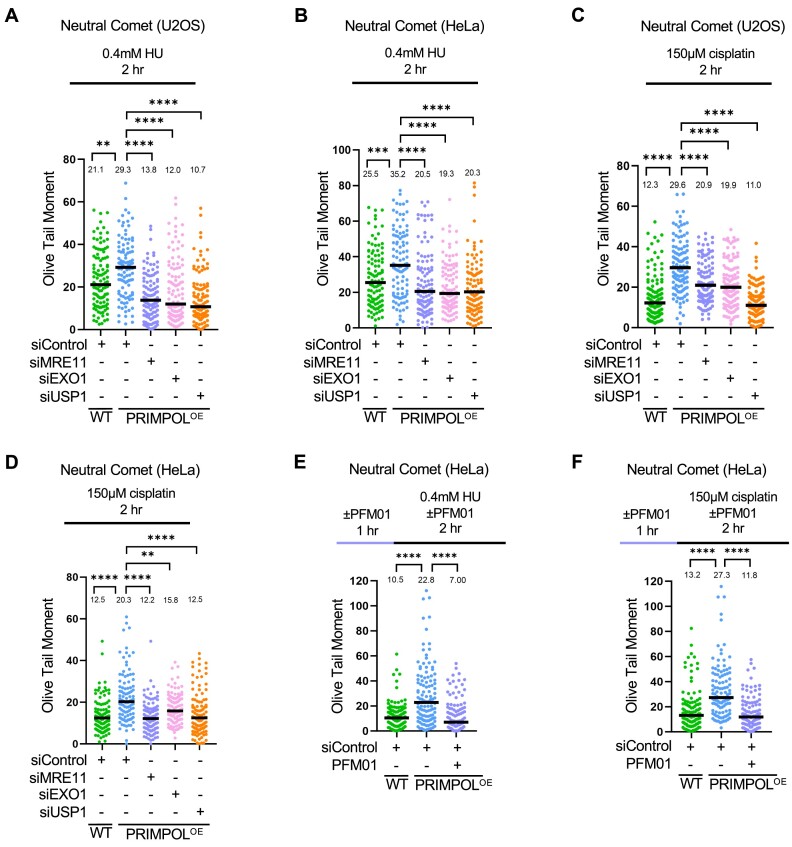
Processing of PRIMPOL-derived ssDNA gaps by MRE11, EXO1 and USP1 leads to DSB formation. (A–D) Neutral comet assays showing that treatment with 0.4 mM HU (**A, B**) or 150μM cisplatin (**C, D**) for 2 h causes accumulation of DSBs in U2OS (A, C) and HeLa (B, D) PRIMPOL-overexpressing cells, which are suppressed by depletion of MRE11, EXO1 or USP1. At least 100 comets were quantified for each sample. The median values are marked on the graph, and asterisks indicate statistical significance (Mann–Whitney, two-tailed). (E, F) Neutral comet assays showing that inhibition of MRE11 endonuclease activity using the specific inhibitor PFM01 (100μM) suppresses DSB accumulation in PRIMPOL-overexpressing HeLa cells upon treatment with 0.4mM HU (**E**) or 150μM cisplatin (**F**) for 2 h. At least 100 comets were quantified for each sample. The median values are marked on the graph, and asterisks indicate statistical significance (Mann–Whitney, two-tailed). Schematic representations of the assay conditions are shown at the top.

We next sought to investigate the consequences of ssDNA gap expansion on genomic stability. To this end, we measured the impact of ssDNA gap processing factors on DSB formation using the neutral comet and γH2AX foci immunofluorescence assays. Neutral comet assays indicated that PRIMPOL-overexpressing HeLa and U2OS cells showed increased DSB accumulation compared to control cells, upon treatment with 0.4mM HU (Figure 6A, B) or 150μM cisplatin (Figure 6C, D) for 2h. These findings argue that ssDNA gaps are ultimately converted to DSBs. Indeed, depletion of MRE11 or of EXO1 suppressed DSB accumulation in PRIMPOL-overexpressing cells (Figure 6A-D), indicating that limiting ssDNA accumulation suppresses DSB formation, and arguing that gap expansion is an essential step in DSB formation. These findings were confirmed by employing γH2AX foci immunofluorescence assays as a readout of DSB formation in HeLa and U2OS PRIMPOL-overexpressing cells (Supplementary Figure S5). Interestingly, DSBs induced in PRIMPOL-overexpressing cells by treatment with 0.4mM HU or 150μM cisplatin were also suppressed upon inhibition of MRE11 endonuclease activity with the specific inhibitor PFM01 (Figure 6E, F), arguing that MRE11 endonuclease activity contributes to the conversion of ssDNA gaps into DSBs.

Finally, in both neutral comet and γH2AX foci immunofluorescence assays, we observed that USP1 depletion suppressed DSB formation induced by HU or cisplatin treatment in PRIMPOL-overexpressing HeLa and U2OS cells (Figure 6A–D, Supplementary Figure S5). Overall, these findings argue that, by promoting PCNA de-ubiquitination, USP1 causes an increase in replication-associated ssDNA gaps which are subsequently converted into DSBs, potentially causing genomic instability.

## Discussion

Recent studies have identified ssDNA gaps as a potential vulnerability to be exploited in cancer therapy (68). In particular, ssDNA gap accumulation was found to better correlate with chemosensitivity in certain BRCA-deficient genetic backgrounds, suggesting that gap suppression may be a mechanism of chemoresistance in these tumors (28,29,39,43,47,69,70). PRIMPOL depletion reduces gap formation in BRCA-deficient cells (26,28,29), highlighting a physiological role for PRIMPOL in gap formation in these cells. In DNA repair proficient backgrounds, increased PRIMPOL expression was previously shown to reduce fork reversal and enhance ssDNA gap formation upon cisplatin treatment (26,29). This likely reflects increased PRIMPOL-mediated repriming as a mechanism of fork restart at the expense of fork reversal. Indeed, our current analysis indicates that gap induction is PRIMPOL-dose dependent (Supplementary Figure S2).

**Figure 7.**
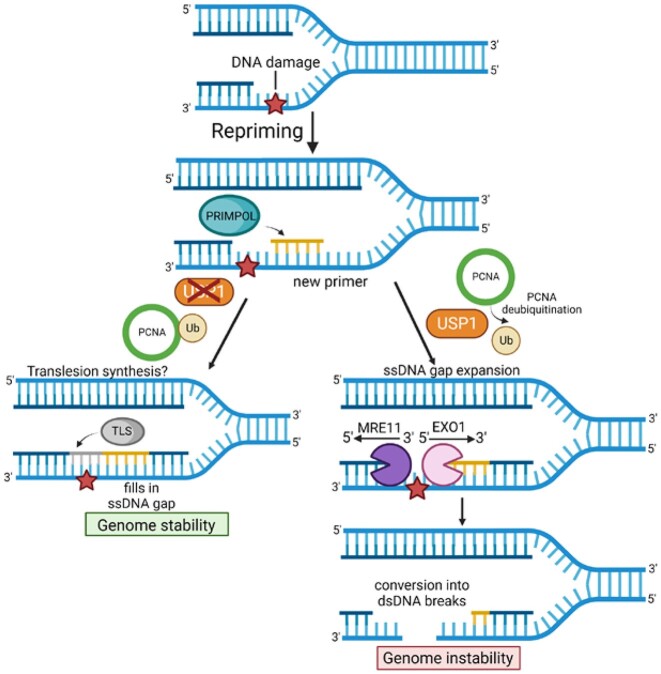
Schematic representation of the proposed model. PRIMPOL-generated gaps are engaged by MRE11 and EXO1, even in BRCA-proficient cells, and expanded bidirectionally. USP1, through de-ubiquitination of PCNA, promotes gap expansion by MRE11 and EXO1, presumably by suppressing TLS-mediated gap filling. Under these conditions, upon extensive processing by MRE11 and EXO1, PRIMPOL-generated gaps can be converted into DSBs. Created with BioRender.com.

Considering the relevance of ssDNA gap accumulation described above, understanding their processing is of significant importance. Here, we show that PRIMPOL-generated gaps are expanded bidirectionally by the MRE11 and EXO1 exonucleases. Previously, MRE11 exonuclease inhibition was shown to suppress ssDNA gap accumulation in BRCA-deficient cells, and it was proposed that BRCA proteins inhibit MRE11 in gap expansion (29). Here, we show that, even in BRCA-proficient cells, MRE11 is able to bind to gapped DNA at the 3′ end of the gap and extend it in the 5′ direction (Figure 7). Thus, our work suggests that BRCA proteins are not entirely suppressing MRE11 engagement on nascent ssDNA gaps and subsequent gap expansion.

To what extent the 5′ end of the gap is also engaged by exonucleases was unclear. Previously, it was shown that loss of EXO1 reduces nascent DNA resection upon treatment with BPDE (27) and UV (71), which induce DNA adducts, or, more recently, upon ATR inhibition (9). These findings may suggest a role for EXO1 in ssDNA gap processing. Indeed, here we show that EXO1 binds to the 5′ end of the gap and extends it in the 3′ direction, in the opposite direction from MRE11. Intriguingly, depletion of either MRE11 or EXO1 fully suppressed gap accumulation in PRIMPOL-overexpressing cells, suggesting that the two nucleases are epistatic, even though they expand the gap in opposite directions. This is in fact reminiscent of their activity in the degradation of reversed forks in BRCA-deficient cells, where depletion of either of them can fully suppress fork degradation (15,20,45). Overall, these findings argue that MRE11 and EXO1 are potentially co-regulated to act in tandem on nascent DNA. Moreover, we also show that inhibition of MRE11 endonuclease activity suppresses DSB accumulation in PRIMPOL-overexpressing cells under ssDNA gap-inducing conditions, potentially suggesting that MRE11 may cleave the intact parental strand at the ssDNA gap site through its endonuclease activity, to generate the DSB.

In this study, we also show that PCNA ubiquitination levels affects the repair of ssDNA gaps during replication. TLS has been previously proposed to maintain continuous replication by limiting ssDNA gaps induced by replication stress (51). More recently, it was shown that in BRCA-deficient cells, PRIMPOL-generated gaps are repaired by a REV1-dependent mechanism which requires RAD18-mediated PCNA ubiquitination (28). Finally, using an assay which measures gap filling behind the replication fork, it was shown that RAD18-mediated PCNA ubiquitination promotes the repair of cisplatin-induced ssDNA gaps generated by PRIMPOL overexpression, during the G2 phase of the cell cycle (29). Here, we show that RAD18 depletion does not increase ssDNA gap accumulation during DNA replication, which is perhaps in line with the role of RAD18 in repairing ssDNA gaps during G2 phase. However, we also show that depletion or inhibition of USP1 suppresses the accumulation of ssDNA gaps during replication. Moreover, we show that this suppression reflects the USP1 de-ubiquitination activity towards PCNA rather than other substrates, since it depends on RAD18-mediated PCNA ubiquitination. Thus, our findings argue that PCNA ubiquitination may play a role in replication-associated gap suppression, at least when de-ubiquitination is perturbed upon the loss of USP1 activity. Moreover, we show that suppression of ssDNA gap accumulation by USP1 inhibition also depends, at least in part, on the TLS polymerase REV1. Overall, these results suggest that PCNA ubiquitination-mediated TLS promotes replication-associated gap filling (Figure 7).

A potential caveat of this study is that it does not include experiments aiming at correcting the USP1 deficiency by exogenous re-expression of wildtype or catalytic-inactive USP1. However, we employed two different siRNA oligonucleotides for USP1 knockdown, which showed identical phenotypes, thus arguing against a non-specific effect. We moreover present similar results using USP1 inhibition by two different small molecule inhibitors. We first employed ML323, which inhibits the USP1–UAF1 interaction. Recent studies have shown that ML323 treatment can also downregulate PCNA expression (72,73). Whether this plays a role in the observed gap suppression phenotype is unclear. Nevertheless, our studies with the second inhibitor, namely the catalytic site inhibitor KSQ-4279, further substantiate the specificity of the USP1 catalytic activity in gap promotion.

While we anticipate that the observed reduction in replication-associated gaps upon loss of USP1 activity reflects an increase in PCNA ubiquitination-mediated TLS (Figure 7), an intriguing alternative explanation may be that PCNA ubiquitination also regulates the engagement of nucleases on DNA in a more direct manner, potentially independent of TLS. We show that loss of USP1 reduces MRE11 and EXO1 binding to nascent DNA at ssDNA gaps. This may be simply explained by a reduction in the number of gaps because of increased TLS-mediated gap filling. However, it may also reflect the alternative explanation that increased PCNA ubiquitination inhibits MRE11 and/or EXO1 recruitment, thus suppressing gap expansion. Indeed, PCNA was previously shown to interact with EXO1 and promote its processivity (74). Whether the ubiquitination of PCNA affects this interaction remains to be investigated.

Finally, we show that, upon expansion by MRE11 and EXO1, PRIMPOL-generated ssDNA gaps are ultimately transformed into DSBs. While the mechanism underlying this process is unclear, these findings argue that, unless rapidly filled during DNA replication, ssDNA gaps have the potential to generate toxic structures and genomic instability, which perhaps explain why ssDNA gap accumulation is associated with chemosensitivity (28,29,39,43,47,69,70). We moreover show that loss of USP1 also suppresses DSB formation upon ssDNA gap accumulation conditions, further demonstrating that gap protection during replication is essential for genomic stability. These findings identify USP1 as a factor which, under certain circumstances, can promote genomic stability. Since it was previously shown that ML323 treatment causes replication stress and cisplatin sensitization in certain cancer cells (64,75), our findings suggest that the development of USP1 inhibitors for their potential use in cancer treatment (57) should be carefully evaluated to take into consideration the impact of the genetic background.

## Supplementary Material

gkad1237_Supplemental_Files

## Data Availability

The source data underlying all figures are provided within this paper, including the values plotted in graphs, the exact *P*-values and the un-cropped blots, which are presented in Supplementary Table S1.
