# Supplementary material for: USP1-dependent nucleolytic expansion of PRIMPOL-generated nascent DNA strand discontinuities during replication stress
Source: Nucleic Acids Res. 2024 Jan 5;52(5):2340–54. doi: 10.1093/nar/gkad1237 (PMC10954467; doi:10.1093/nar/gkad1237)
Supplement: gkad1237_Supplemental_Files [file gkad1237_supplemental_files.zip › Supplementary Material.pdf]

SUPPLEMENTARY MATERIAL

Legends to Supplementary Tables

Supplementary Table S1. The source data underlying each of the main and supplementary figure panels, including: the values plotted in graphs, the exact p-values, and the uncropped blots.

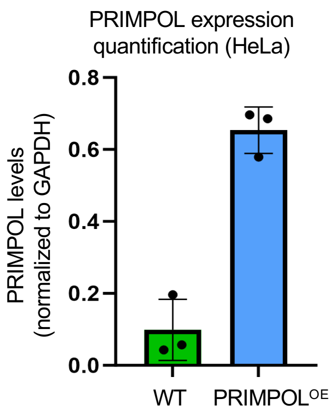

Supplementary Figure S1. Characterization of the PRIMPOL overexpression cell system used.

Quantification of PRIMPOL protein levels in wildtype and PRIMPOL-overexpressing HeLa cells from three independent western blot experiments. Band intensities were quantified using ImageJ. PRIMPOL levels were normalized to GAPDH levels.

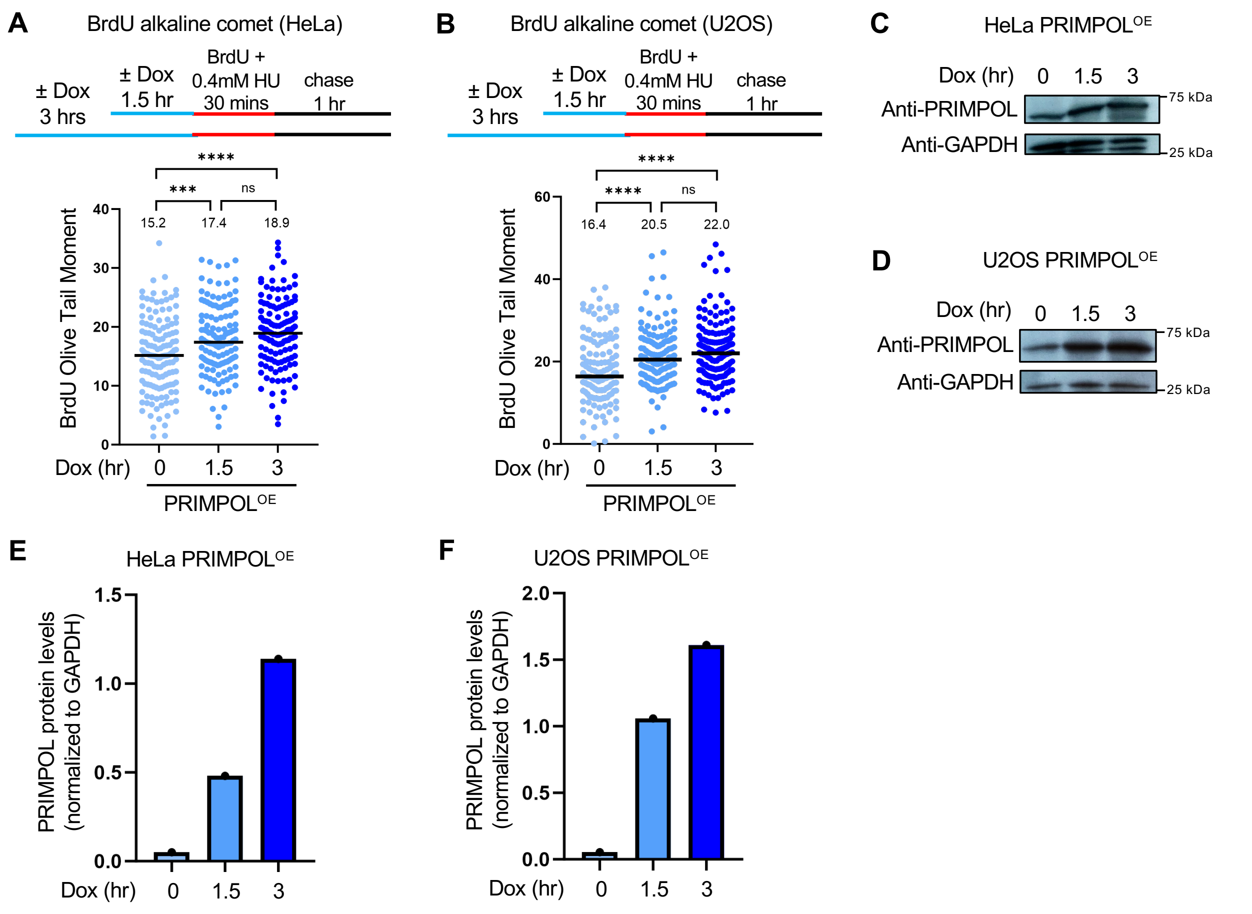

Supplementary Figure S2. PRIMPOL expression results in gap accumulation in a dose-dependent manner.

A,B. BrdU alkaline comet assays showing that increasing PRIMPOL expression in HeLa (**A**) and U2OS (**B**) cells results in higher levels of ssDNA gaps upon treatment with 0.4mM HU. At least 130 nuclei were quantified for each condition. The median values are marked on the graph and listed at the top. Asterisks indicate statistical significance (Mann-Whitney, two-tailed). Schematic representations of the assay conditions are shown at the top.

C,D. Western blots showing PRIMPOL protein levels in HeLa (**C**) and U2OS (**D**) cells under the experimental conditions employed above.

E,F. Quantifications of PRIMPOL protein levels in HeLa (**E**) and U2OS (**F**) cells under the experimental conditions employed above. Western blot band intensities were quantified using ImageJ. PRIMPOL levels were normalized to GAPDH levels.

These experiments were performed in HeLa and U2OS cells expressing a doxycycline-inducible PRIMPOL construct. To obtain the inducible cell lines, HeLa and U2OS cells were consecutively transduced with pLV[Exp]-Bsd-CMV>rtTA and pLV[Exp]-Hygro-TRE>hPRIMPOL lentiviral constructs (Cyagen). To induce PRIMPOL expression, cells were treated with 6.7μg/ml doxycycline for the indicated periods of time.

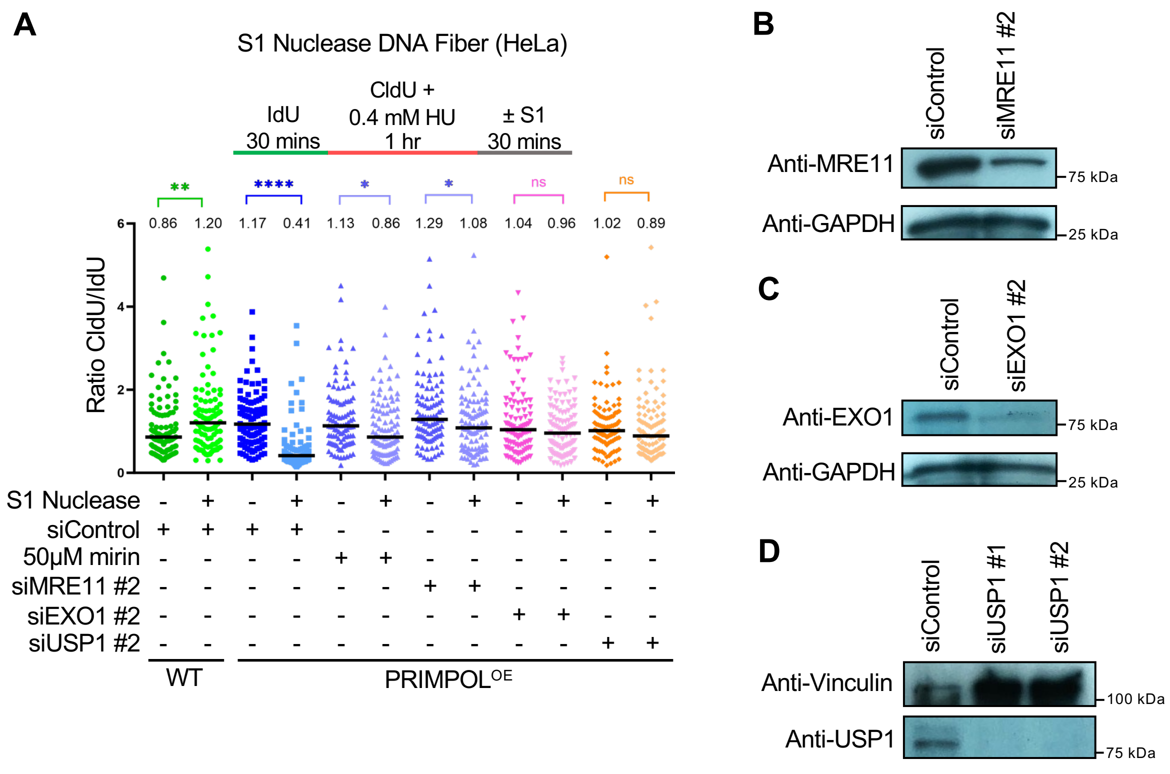

Supplementary Figure S3. Impact of EXO1, MRE11 and USP1 on gap formation in PRIMPOL-overexpressing cells.

A. S1 nuclease DNA fiber combing assay showing suppression of ssDNA gap accumulation in HeLa PRIMPOL-overexpressing cells treated with 0.4mM HU upon depletion of EXO1, MRE11 or USP1 using a second independent siRNA oligonucleotide for each of these factors. The ratio of CldU to IdU tract lengths is presented, with the median values marked on the graphs and listed at the top. At least 100 tracts were quantified for each sample. Asterisks indicate statistical significance (Mann-Whitney, two-tailed). A schematic representation of the assay conditions is shown at the top. To inhibit MRE11 exonuclease activity, 50μM mirin was added during the labeling with IdU and CldU.

B-D. Western blots showing siRNA-mediated knockdown of MRE11 (**B**), EXO1 (**C**) and USP1 (**D**) in HeLa PRIMPOL-overexpressing cells using the second independent siRNA oligonucleotide employed above.

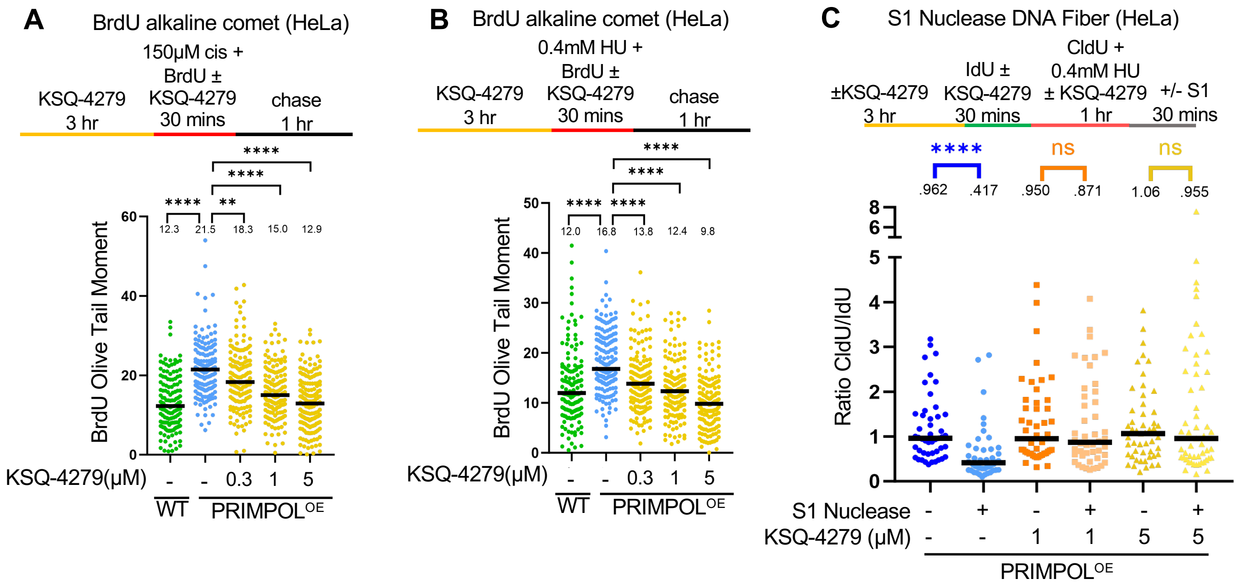

Supplementary Figure S4. Inhibition of USP1 de-ubiquitination activity by KSQ-4279 suppresses the accumulation of ssDNA gaps in PRIMPOL-overexpressing cells.

A,B. BrdU alkaline comet assay showing that inhibition of USP1 by treatment with KSQ-4279 as indicated, suppresses the accumulation of replication-associated ssDNA gaps induced by exposure to 150μM cisplatin (**A**) or 0.4mM HU (**B**) in HeLa PRIMPOL-overexpressing cells. At least 135 nuclei were quantified for each condition. The median values are marked on the graph and listed at the top. Asterisks indicate statistical significance (Mann-Whitney, two-tailed). Schematic representations of the assay conditions are shown at the top.

C. S1 nuclease DNA fiber combing assay showing that inhibition of USP1 by treatment with KSQ-4279 as indicated suppresses the accumulation of replication-associated ssDNA gaps induced by exposure to 0.4mM HU in HeLa PRIMPOL-overexpressing cells. The ratio of CldU to IdU tract lengths is presented, with the median values marked on the graphs and listed at the top. At least 45 tracts were quantified for each sample. Asterisks indicate statistical significance (Mann-Whitney, two-tailed). A schematic representation of the assay conditions is shown at the top.

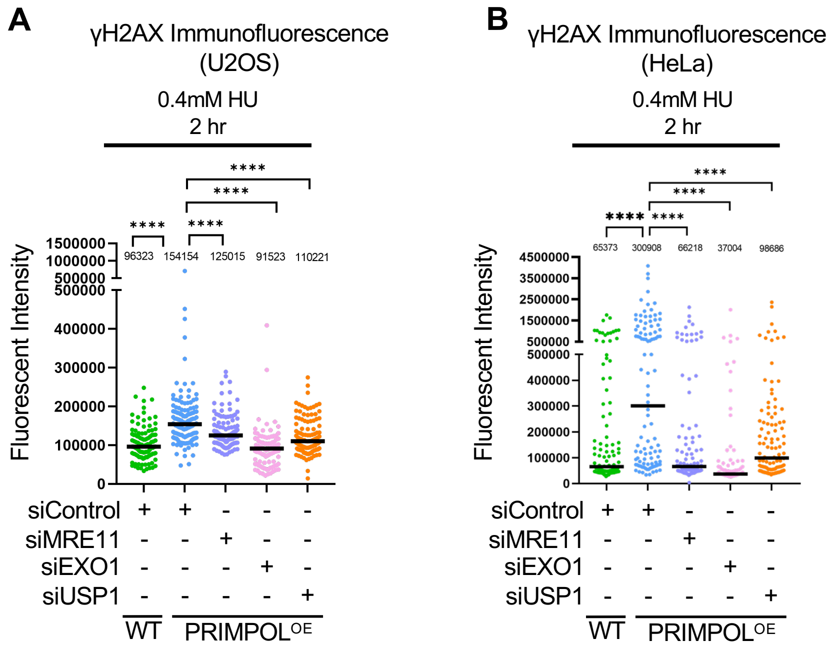

Supplementary Figure S5. **Processing of PRIMPOL-derived ssDNA gaps into DSBs.**

γH2AX immunofluorescence showing that that treatment with 0.4mM HU for 2 hours increases γH2AX foci in in U2OS (**A**) and HeLa (**B**) PRIMPOL-overexpressing cells, which are suppressed by depletion of MRE11, EXO1, or USP1. At least 100 cells were quantified for each condition. The median value is represented on the graphs, and asterisks indicate statistical significance (Mann-Whitney, two-tailed).
